# Alleviate Similar Object in Visual Tracking via Online Learning Interference-Target Spatial Structure

**DOI:** 10.3390/s17102382

**Published:** 2017-10-19

**Authors:** Guokai Shi, Tingfa Xu, Jiqiang Luo, Jie Guo, Zishu Zhao

**Affiliations:** 1 School of Optoelectronics, Image Engineering & Video Technology Lab, Beijing Institute of Technology, Beijing 100081, China; shi_guokai_123@126.com (G.S.); luojiqiang@yeah.net (J.L.); jieguo_2013@163.com (J.G.); nicholasldm@126.com (Z.Z.); 2 Key Laboratory of Photoelectronic Imaging Technology and System, Ministry of Education of China, Beijing 100081, China

**Keywords:** similar object interference, correlation filter based trackers, online structured learning

## Abstract

Correlation Filter (CF) based trackers have demonstrated superior performance to many complex scenes in smart and autonomous systems, but similar object interference is still a challenge. When the target is occluded by a similar object, they not only have similar appearance feature but also are in same surrounding context. Existing CF tracking models only consider the target’s appearance information and its surrounding context, and have insufficient discrimination to address the problem. We propose an approach that integrates interference-target spatial structure (ITSS) constraints into existing CF model to alleviate similar object interference. Our approach manages a dynamic graph of ITSS online, and jointly learns the target appearance model, similar object appearance model and the spatial structure between them to improve the discrimination between the target and a similar object. Experimental results on large benchmark datasets OTB-2013 and OTB-2015 show that the proposed approach achieves state-of-the-art performance.

## 1. Introduction

Research interest in visual object tracking comes from the fact that it is widely used in smart and autonomous systems, e.g., anomaly detection, smart video compression, and driver intelligent assistance systems. The main challenges of visual tracking are how the tracker can online adapt to the large appearance variations including occlusion, abrupt motion, deformation, illumination variations, in plane rotation, out of plane rotation, similar object interference, etc.

To get a more general adaptive tracker, researchers have proposed many tracking approaches using various visual representations. These approaches, which are mainly focused on the research of the object appearance model, can be divided into two categories: generative models and discriminative models. Generative models [1,2,3,4,5] search the closest description in model space as the target observation to estimate target state. These models adopt an appearance model to describe the target appearance state without considering the background information of the target effectively. Therefore, they have low discrimination when scene is complex. Compared with generative models, discriminative models [6,7,8] have better discrimination and generalization ability. These models formulate object tracking as a binary classification problem that does not estimate target specific location directly, and their accuracy is limited by the number of candidate tests.

Recently, Correlation Filter (CF) based trackers have gained much attention in visual tracking, and exhibited outstanding performance in both speed and accuracy. These trackers can be performed effectively by employing all circular shifts of the positive sample in the Fourier domain, and overcome the shortcomings of traditional discriminative model using densely-sampled samples. Bolme et al. [9] introduced CF into visual tracking for the first time. Henriques et al. [10] demonstrated the connection between Ridge Regression with cyclically shifted samples and classical correlation filters. Then, the CF tracking model was extended by its kernelized version (KCF) [11] to handle high-dimensional features. By learning the scale model of the target using one-dimensional correlation filters, the DSST proposed in [12] overcame the target scale change. Danelljan et al. [13] introduced a spatial regularization into CF tracking model to deal with the boundary effects of circularly shifted patches. Ma et al. [14] applied random ferns detector to CF tracking framework, and developed a long-term correlation tracking to solve serious occlusion. In [15], a generic formulation was proposed for jointly learning the filter and target response to alleviate motion blur and fast motion. Moreover, Bertinetto et al. [16] integrated the advantages of DSST [12] and DAT [17] effectively, and proposed complementary learner called Staple to alleviate target deformation problem. Thus far, CF trackers have alleviated some common problems effectively in visual tracking, e.g., occlusion [14], large scale variations [12], deformation [16,17], out-of-plane object rotation [16,17], fast motion [15] and motion blur [18].

However, similar object interference is still a challenge in visual tracking, and has no corresponding algorithm to solve this problem in CF tracking framework. Most existing tracking approaches, which are used to deal with occlusion problem, have difficulty discriminating the target from a similar object. In [5,8,14,19], occlusion detection mechanism was introduced into tracking framework to regain lost target. In [20,21], the appropriate historical tracking model was chosen to correct the drift after the occlusion. In [22,23], part-based tracking strategy was applied to CF tracking framework to overcome the occlusion. In [24], multiple trackers based on different feature representations were integrated within a probabilistic framework to alleviate the occlusion. In [25], a CF model with an anisotropic response was constructed for dealing with the occlusion. The shortcoming of these algorithms is that there is no cooperative consideration of the association information between the target and the similar target. When the target is occluded by a similar object, the target and the similar object not only have similar appearance features but also are in same surrounding context. Therefore, only using the information of the target is not sufficient for discrimination.

Based on above discussions, we propose an approach that jointly learns the appearance model of the target, the appearance of similar objects and the spatial structure between them to alleviate the interference from similar objects. In the proposed approach, we train the target and similar objects by their own appearance model. When the target is occluded by a similar object, the occlusion object is matched by its own appearance model, which avoids the appearance model of the target being updated by the similar object.

Our main contributions are listed as follows.

(1) We propose to add ITSS constraint into existing CF tracking model for alleviating similar object interference. The proposed approach jointly learns the target appearance model, similar object appearance model and the spatial structure between the target and similar objects.
(2) We propose to introduce interference degree weight into the model, which makes our approach switch between the baseline model and the constraint model (integrating the baseline model and ITSS constraint) dynamically.
(3) During the model update, instead of only updating the target appearance model, we collaboratively update the target model and the interference object model. Moreover, we propose combining the target model and the interference model for re-detecting the lost target when the target is almost totally occluded by a similar object.

The rest of this paper is organized as follows. Section 2 presents the proposed method. Simulation results are presented in Section 3, and conclusions are drawn in Section 4.

## 2. Proposed Approach

We found that the spatial structure constructed by similar object and target is very useful information that can help to distinguish target from similar objects effectively. When the target and a similar object overlap, we can effectively identify the occlusion relationship between the target and a similar object by their spatial structure. Once the spatial structure is obtained, the model update can be controlled reasonably to alleviate occlusion of similar target. Thus, we propose utilizing online learning interference-target spatial structure constraint for alleviating similar object occlusion.

**Figure 1:**
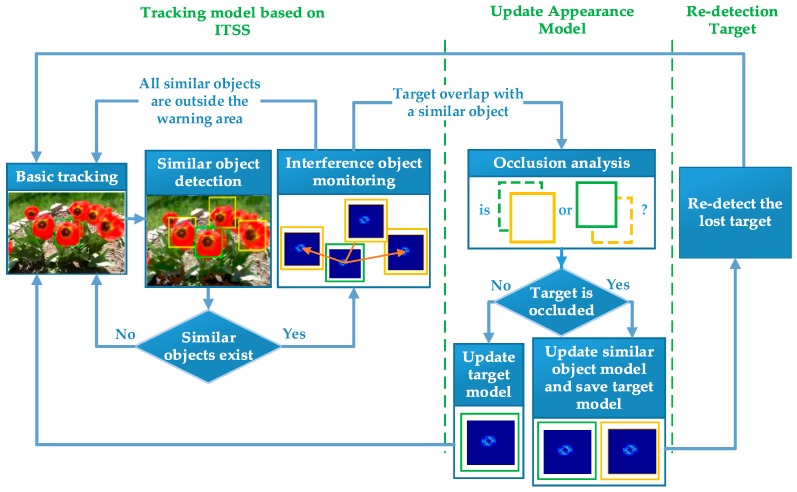
The flowchart of the proposed approach.

Figure 1 shows the flowchart of the proposed approach. We divide the approach into three stages: tracking based on ITSS model, update appearance model and re-detection the target. The similar object detected will be regarded as the potential interference object which is used to construct interference-target spatial structure graph. The edges in the graph reflect the spatial structure between the target and each similar object, and can be used to determine whether the target overlaps with a similar object. The graph’s nodes describe the target’s and similar object’s appearance models. If there exists overlapping between the target and a similar object, we will use our occlusion analysis strategy to determine whether the target is occluded by the similar object. Then, we accord the result of the occlusion analysis to update models including the target model and similar object model. If the target is severely or totally occluded, the flow will be into re-detection stage to detect the lost target. If all the similar objects are outside the warning area in a frame, the process will switch to the baseline tracker.

### 2.1. Detecting Potential Interference

The interference from a similar object is gradually formed when similar objects get closer to the target. Therefore, we assume that there exists a warning area where similar object is considered as the potential interference. The warning area is divided into eight overlapping search regions. Each search region has a partial overlap with the target, but the size of the overlapping area is controlled less than half the size of the target. Similar targets are detected simultaneously by parallel computing in the eight search regions. The spatial layout has several advantages. First, the maximum response of the detector will be not on the target when similar object has attended in the search area. Second, each search area is formulated conveniently by the location and size of the target, and the eight search areas can be proportionally synchronized when the target size changes.

Usually, the similar object and target will deform during tracking, and the degree of the deformation is random. To overcome the problem, we consider two types of features, one is sensitive to deformation and the other is insensitive. Inspired by Staple tracker [16], we integrate a global color histogram and HOG features to train detector for detecting similar object. Our method is different from Staple tracker as follows. First, the detector model only models the target appearance using correlation filters without including any surrounding context. Second, our method is not a simple combination of two types of features by a fixed weight but dividing the detection process into coarse detection and fine detection. This strategy is beneficial since similar object detection is different from target tracking. Target tracking assumes that the target has existed in the search region. The assumption is invalid in object detection, because if the object is not in the search region, the location of maximal correlation response is not the location of the object.

Specifically, during coarse detection, we take the location of maximal correlation response as the center of a similar object. The correlation response is obtained by the correlation between each search region and the detector trained by HOG features extracted from the estimated target. The confidence map constructed from the correlation response reflects the structural similarity between the estimated target and the testing sample from the search area. The second stage is called fine detection that gains reliable similar objects and eliminates unreliable ones from potential candidate similar objects. In this stage, the color statistical feature is extracted by a color statistical model trained by current estimated target and surrounding context. For more details of the color statistical model training, we refer to [17]. The difference is that we only extract the color feature in each potential target region which does not include any surrounding context. Then, we estimate the similarity between the target and each potential similar object by comparing the similarity of their color histogram.

### 2.2. Interference-Target Spatial Structure Constraint

Zhang [26] proposed tracking multi-target by preserving structural model. Motivated by this approach, we dynamically maintain the spatial structure between the target and a similar object for online supervising them. The purpose is to distinguish the similar object and the target when they occlude each other. Different from [26], we estimate the score of each object appearance (including similar objects and the target) by CF model which takes advantage of surrounding context to improve discrimination efficiently. Moreover, we introduce the weight constraint into the model to reflect the differences of the interference degree from each similar object.

#### 2.2.1. The Score Function of Spatial Structure Model

Interference-target Spatial Structure (ITSS) can be viewed as a topological graph figuratively. We define a graph **G** = (***V***, **E**). Node set ***V*** is constructed by the target appearance and all similar object appearance. The edges set **E** include all connection between the target and each similar object. The edges of **E** can be viewed as springs that constraint between the target and each similar object. The length of the edge indicates the relative distance between the target and each similar object. Meanwhile, the thickness of the edge means the strength of a similar object interferes with the target. Now, our aim is to define a score function for describing the structure of graph **G** and design an optimization method to obtain the structural parameters when the score function reaches a maximum value.

The score function of the configuration $C^{t}=\left\{B_{0}^{t},B_{1}^{t},\ldots ,B_{\left|V-1\right|}^{t},\right\}$ is defined as

(1) $$T\left(C^{t};I^{t},\Theta \right)=\underset{c_{0},h_{0}}{\sum }\langle \alpha_{0},\Phi_{0}(I^{t},B_{c_{0},h_{0}}^{t})\rangle +\overset{\left|V\right|}{\underset{j=1}{\sum }}w_{j}^{t-1}\left(\underset{c_{0},h_{0}}{\sum }\langle \alpha_{j},\Phi_{s}(I^{t},B_{c_{j},h_{j}}^{t})\rangle +\lambda \langle \beta_{j},\Psi_{j}(p_{j}^{t},p_{0}^{t})\rangle \right)$$

The variable $c_{j}$ and $h_{j}$ represent row and column circular shifts from the bounding box of *j*-th similar object in frame $I^{t}$. The variable $B_{c_{j,}h_{j}}^{t}=\left\{P_{c_{j,}h_{j}}^{t},W_{j}^{t},H_{j}^{t}\right\}$ denotes the bounding box of corresponding circular shifts from *j*-th similar object in frame $I^{t}$. $W_{j}^{t},H_{j}^{t}\;$and vector $P_{c_{j,}h_{j}}^{t}\;$denote the width, the high and the center pixel coordinate of bounding box $B_{c_{j,}h_{j}}^{t}$, respectively. The vector $\Phi_{0}$ and $\Phi_{s}\;$denote the feature of the target and similar object. Specifically, when there are no similar objects around the target, $\Phi_{0}\;$is corresponding to the feature used in base tracker. Contrarily, if there are similar objects around the target, $\Phi_{0}$ and $\Phi_{s}\;$are both HOG feature. Moreover, the vector $\Psi_{j}\left(p_{j}^{t},p_{0}^{t}\right)=p_{j}^{t}-p_{0}^{t}$, which is a 2D vector including two attributes of size and direction, denotes the relative location between the *j*-th similar object and the target. The parameter Θ is denoted as $\Theta =\left\{\alpha_{0},\alpha_{1},\ldots ,\alpha_{\left|V\right|-1},\beta_{1},\ldots ,\alpha_{\left|V\right|-1},\right\}$, and |V| is node count in G. We use the distance between each similar object and the target to represent the degree of interference. The closer the distance is, the severer the interference is. This relationship is represented as

(2) $$w_{j}^{t}=\{\begin{array}{ccc} exp\left(-\|\Psi_{j}{\left(p_{j}^{t},p_{0}^{t})\right\|}^{2}\right), & \|\Psi_{j}{\left(p_{j}^{t},p_{0}^{t})\right\|}^{2}<T & \\ & & j\in \left\{0,1,2,\ldots ,\left|V\right|\right\}\\ 0, & \|\Psi_{j}{\left(p_{j}^{t},p_{0}^{t})\right\|}^{2}>T & \end{array}$$

T, which is a constant determined by the size of the searching region and the position relative to a target, is the max-distance between the similar object and the target.

These weight parameters introduced into Equation (1) have two important functions. (1) They control the importance of the corresponding similar object and reflect the interference degree of the similar object to the target. The greater the weight is, the more serious the disturbance is. (2) These weights also maintain a fixed function structure of Equation (1). We do not need to change the form of Equation (1) when the number of similar targets in the warning area changes.

#### 2.2.2. Online Learning for Structured Prediction

We train the parameter Θ by minimizing the mixture loss function L(Θ;C). The score of configuration Equation (1) is rewritten into two components including appearance and deformation cost. We define L(Θ;C) as

(3) $$L\left(\Theta ;C\right)=\ell_{a}\left(\alpha ;I,C\right)+\lambda \ell_{b}\left(\beta ;I,C\right)+\frac{1}{2}{\left\|\Theta \right\|}_{2}^{2}.$$

Here, $\ell_{a}$ is the cost of appearance component

(4) $$\ell_{a}\left(\alpha ;I,C\right)=A_{0}\left(\alpha_{0},I,B_{0}\right)+\overset{\left|V\right|}{\underset{j=1}{\sum }}w_{j}A_{j}\left(\alpha_{j},I,B_{j}\right)\;,$$

and $\ell_{a}$ consists of two parts, the target appearance loss $A_{0}$ and the loss $A_{j}$ of similar object appearance

(5) $$A_{0}\left(\alpha_{0},I,B_{0}\right)=\underset{c_{0},h_{0}}{\sum }\left(\langle \alpha_{0},\Phi_{0}(I,B_{c_{0},h_{0}})\rangle -y_{c_{0},h_{0}}\right)+\mu \|\alpha_{0}\|{}_{2}^{2}$$

(6) $$A_{j}\left(\alpha_{j},I,B_{j}\right)=\underset{c_{j},h_{j}}{\sum }\left(\langle \alpha_{j},\Phi_{s}(I,B_{c_{j},h_{j}})\rangle -y_{c_{j},h_{j}}\right)+\mu \|\alpha_{j}\|{}_{2}^{2}$$

The structure labels $y_{c_{j},h_{j}}\;$and $y_{c_{0},h_{0}}$ are computed by two-dimensional Gaussian function.

The function $\ell_{b}$ is the deformation cost constraint which captures the special structure between the similar object and the target. Mathematically, this amount is described as

(7) $$\ell_{b}\left(\beta ;I,C\right)=\underset{\hat{C}}{\max}\left[S\left(\hat{C};I,\beta \right)-S\left(C;I,\beta \right)+\Delta \left(C,\hat{C}\right)\right]\;.$$

Here, S(C;I,β) is deformation score function described as

(8) $$S\left(C;I,\beta \right)=\overset{\left|V\right|-1}{\underset{j=1}{\sum }}\langle \beta_{j},\Psi (P_{0},P_{j})\rangle$$

Herein, the task-loss $\Delta \left(C,\hat{C}\right)$ is defined based on the amount of overlap between the correct configuration C and the incorrect configuration $\hat{C}$.

(9) $$\Delta \left(C,\hat{C}\right)=\overset{\left|V\right|}{\underset{j=1}{\sum }}\left(1-\frac{\beta_{j}\cap^{\text{​}}{\hat{\beta }}_{j}}{\beta_{j}\cup^{\text{​}}{\hat{\beta }}_{j}}\right)$$

After the transformation above, the problem is how to optimize Equation (3) for learning parameter Θ. Two components α and β from Θ are divided into the first term and the second term of Equation (3). Therefore, we can optimize α and β alternatively by Equations (4) and Equation (7). First, as far as α is concerned, Equation (4) is a linear combination of Equations (5) and (6) which are both conventional correlation filtering operation. We can optimize them by kernel trick and circulant matrix. Conversely, when α is fixed, we can get the solution of β by optimizing Equation (7). Thus, calculating parameter β is transformed to a structured SVM problem. Equation (7) is the maximum of a set of affine functions and does not contain quadratic terms. Thus, it is a convex function. We use a similar method with Pegasos-based algorithm [27] to optimize the problem.

#### 2.2.3. Target Detection with Spatial Constraints

The detection task is to find a configuration with the best score over all possible configurations which maximizes Equation (1) when the model parameter Θ is given in new frame. We first construct a minimum spanning tree model to build the graph G which determines the edge joint between the target and each similar object. The root of the tree is set to the estimated target. Then, we search for a set of connections to construct the tree. Once the structure of the graph is obtained, we find the best score by dynamic programming Equation (10).

(10) $$ℑ\left(\hat{C};i\right)=\underset{j\in child\left\{i\right\}}{\sum }\underset{j}{\max}\left\{ℑ\left(\hat{C};j\right)\right\}+w_{i}\left(\underset{c_{i},h_{i}}{\sum }\langle \alpha_{i},\;\Phi_{s}\left(I,B_{c_{i},h_{i}}\right)\rangle +\underset{i}{\sum }\langle \beta_{i},\;\Psi ({Ρ}_{i},{Ρ}_{0})\rangle \right).$$

All parameters in Equation (10) have the same physical meaning as in Equation (1). The score of the best configuration from this recursive form corresponds to the max score of Equation (1), e.g., $T\left(\hat{C}\right)=ℑ\left(\hat{C};0\right)$. The algorithm automatically finds a best configuration for the target and every similar object.

### 2.3. Update Model

Our model update method is controlled by the occlusion estimation, and the model is updated when there is no occlusion. In our approach, the occlusion estimation is divided into two steps: occlusion detection and occlusion identification.

#### 2.3.1. Occlusion Detection

We dynamically maintain a topological graph, which consists of similar objects and the target, by the method proposed in Section 2.2. The relative positive vector between the target and each similar object is equivalent to provide us with an identifier that identifies the target and the similar object. We estimate the occlusion between $B_{i}$ and $B_{0}$ by the overlap between them when $w_{i}^{t}=max\left\{w_{j}^{t}|j=1,2,\ldots ,\left|V\right|-1\right\}$. The overlap is estimated by Equation (11).

(11) $$f\left(m\right)=\|P_{i}^{t}\left(m\right)-P_{0}^{t}{\left(m)\right\|}_{2}^{2}-\frac{1}{2}\left(B_{i}^{t}\left(m\right)+B_{0}^{t}\left(m\right)\right)$$

By the Equation (11), we can compute the positional relation of abscissa and ordinate between $B_{i}$ and $B_{0}$, respectively. We use f(x) to represent the positional relation of abscissa between the two image areas. In this case, $\|P_{i}^{t}\left(x\right)-P_{0}^{t}{\left(x)\right\|}_{2}^{2}$ represents the distance of abscissa between center point $P_{i}^{t}$ and $P_{0}^{t}$. $B_{i}^{t}\left(x\right)+B_{0}^{t}\left(x\right)$ represents the sum of the width of $B_{i}$ and $B_{0}$. Likewise, we utilize f(y) to represent the positional relation of ordinate between $B_{i}$ and $B_{0}$.

#### 2.3.2. Occlusion Identification

If f(x)<0 and f(y)<0, there exists overlap between $B_{i}$ and $B_{0}$. We need to figure out whether $B_{0}$ is occluded by $B_{i}$ or not. The occluded object has more significant changes in appearance feature than the not occluded object. Hence, corresponding confidence response map will show more dramatic changes. To reduce the interference information from surrounding context, we only estimate the confidence response map corresponding to $B_{i}$ and $B_{0}$, in which background information is not included. We use the combination [19] of the average peak-to-correlation energy $E_{i}$ and $E_{0}$ and maximum response score of the response map $R_{i,max}$ and $R_{0,max}$ to estimate the change of both the confidence maps.

(12) $$E=\frac{{\left|R_{max}-R_{min}\right|}^{2}}{mean{\left(\sum_{w,h}\left(R_{w,h}-R_{min}\right)\right)}^{2}}$$

We compare $R_{i}$, $R_{0}$, $E_{i}$ and $E_{0}$ in the current frame with their respective historical average values $R_{i}^{ha}$, $R_{0}^{ha}$, $E_{i}^{ha}$ and $E_{0}^{ha}$. These four parameters are estimated before the overlap between $B_{i}$ and $B_{0}$, which are considered as the feedback from non-polluting tracking results. If $R_{0}/R_{0}^{ha}<R_{i}/R_{i}^{ha}$ and $E_{0}/E_{0}^{ha}<E_{i}/E_{i}^{ha}$, the target $B_{0}$ is occluded by the similar object $B_{i}$. Otherwise it is contrary.

Next, we can update the appearance model of the target and the similar object according to the result of the occlusion detection. We optimize these models by kernel trick and circulant matrix, so our model update formula is defined as Equation (13).

(13) $${\hat{\alpha }}_{i,t}^{d}=\eta \frac{\hat{y}⊙{\hat{\Phi }}_{i,t}^{d}}{\sum_{j=1}^{D}{\hat{\Phi }}_{i,t}^{j}⊙{\bar{\Phi }}_{i,t}^{j}+\lambda }+\left(1-\eta \right){\hat{\alpha }}_{i,t-1\;}$$

Here, ⋀ represents the Fourier Transform. The superscript d corresponds to a dimensional component of HOG feature. The subscript i is the appearance model identifier, e.g., i = 0 represents object appearance model. η is a learning rate fixed to 0.075, and t denotes the *t*-th frame. The bar—represents complex conjugation.

### 2.4. Target Re-Detection

When the target is almost completely occluded by a similar object, the distance between the target and the occlusion object $\|\Psi {\left({Ρ}_{i},{Ρ}_{0})\right\|}_{2}^{2}$ will become very small. In this case, ITSS constraint will lose its effectiveness. To avoid the target being erroneously positioned on the occlusion object, we collaboratively employ the target appearance model and similar object appearance model to re-detect the lost target. Specifically, the re-detection strategy is divided into three parts: detection model, startup condition and termination condition.

#### 2.4.1. Detection Model

When f(x)=0 and f(y)=0, $B_{i}$ and $B_{0}\;$are both in the edge position of their overlapping and non-overlapping area. Currently, $B_{i}$ and $B_{0}$ are believed to be reliable and free from contamination from each other. We save the appearance model $D_{0}$ trained by $B_{0}$. $D_{i}$ and $D_{0}$ will be used to detect the $B_{0}$, when $B_{0}$ is lost for the occlusion of $B_{i}$. Specifically, our approach tracks $B_{i}$ and updates its model online, and detects $B_{0}$ in surrounding context of $B_{i}$.

#### 2.4.2. Startup Condition

We activate the re-detection mechanism when the target is lost. In our tracking framework, the spatial structure constraint can alleviate the part occlusion. However, when $B_{0}$ is almost completely occluded by $B_{i}$, the max values of the two response maps from tracking $B_{i}$ and $B_{0}$ will point to nearly the same location in $B_{i}$, and the spatial structure constraint becomes disabled. We define this criterion according to Equation (14).

(14) $$\|\Psi {\left({Ρ}_{i},{Ρ}_{0})\right\|}_{2}^{2}<\frac{1}{6}\sqrt{width{\left(B_{i}\right)}^{2}+height{\left(B_{i}\right)}^{2}}$$

When Equation (14) is established, we need to activate re-detection strategy to detect the lost target.

#### 2.4.3. Termination Condition

If $R_{0}>k_{R}R_{0}^{history}$ and $E_{0}>k_{E}E_{0}^{history}$, we consider the detection result reliable and then terminate detection and switch task to baseline tracker. Here, $k_{R}$ and $k_{E}$ are predefined ratios. In this paper, the two ratios are 0.65 and 0.42, respectively.

## 3. Experiments

Our experimental observations are reported from two aspects: special performance evaluation and comprehensive performance evaluation. In special performance evaluation, we evaluate the proposed tracking algorithm and other six state-of-the-art tracking algorithms on nine challenging sequences including occlusion from a similar object to demonstrate the effectiveness of the proposed approach for alleviating similar object interference. In comprehensive performance evaluation, the goal is to demonstrate the comprehensive performance of the proposed algorithms in various challenges including serious occlusion, noise disturbance, non-rigid shape deformation, out-of-plane object rotation, pose variation, similar object interference, etc.

To achieve a latest comparison, we report the results of several recent trackers by adding these trackers to the testing framework of visual tracking benchmark. In experiments, each tracker uses the source code provided by the author. The parameter settings from authors are kept for all the test sequences. All testing sequences are from the OTB-2015.

### 3.1. Baseline Tracker

The Staple tracker combines template and histogram scores to alleviate deformation, and significantly outperforms many state-of-the-art trackers in comprehensive performance. However, color histogram weakens structural information while alleviates deformation, which leads to the poor performance of Staple tracker when there exists similar object in the scenes. Thus, we choose the Staple tracking algorithms as the baseline tracker. The new tracker integrates the basic tracker and the proposed ITSS.

### 3.2. Specific Performance Evaluation

To fully reflect the merits of our approach, we evaluate the specific performance of our approach from two aspects: qualitative evaluation and quantitative evaluation.

#### 3.2.1. Qualitative Evaluation

**Figure 2:**
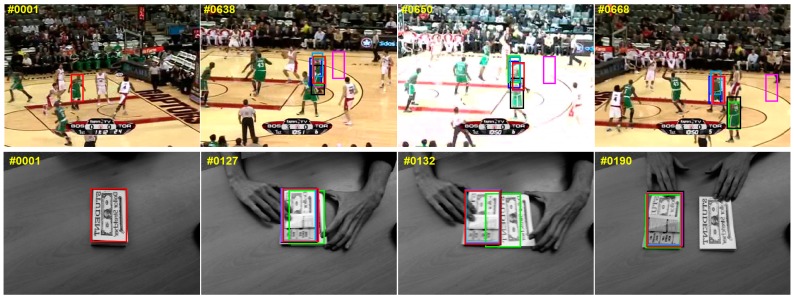

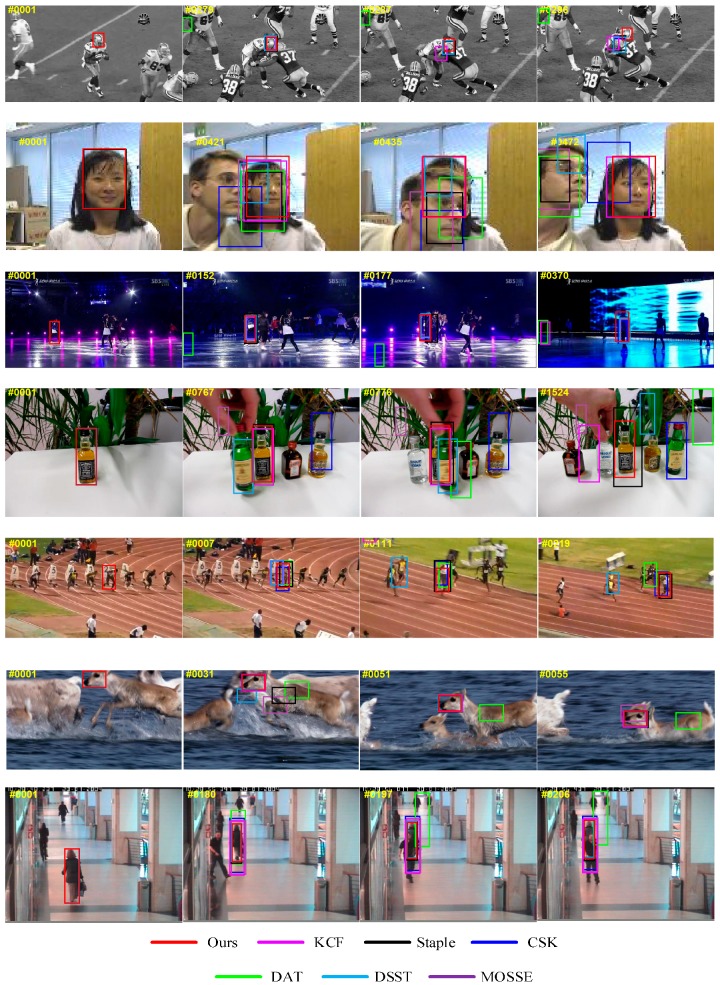
Qualitative comparisons. We list the tracking results of the representative frames of nine sequences (*Basketball*, *Coupon*, *Football*, *Girl*, *Shaking1*, *Liquor*, *Bolt2*, *Deer* and *Walking2* from top to bottom) for seven trackers, and our results are marked with red solid rectangles.

Figure 2 reports the qualitative evaluation results divided into four columns from left to right in each row. The first column is the initial frame where the target is marked by red solid rectangle. The second column is a frame before occlusion occurs. The third column is the frame where there is occlusion between the target and similar objects. The fourth column indicates that the occlusion is removed. The qualitative evaluation is carried out by comparing these four columns in each sequence. We compare the proposed approach with six state-of-the-art trackers: CSK [10], DAT [17], KCF [11], Staple [16], MOSSE [9] and DSST [12]. To demonstrate the effectiveness of the proposed algorithm, we select nine typical sequences. These sequences contain some objects that are not only similar in color but also similar in structure to the target, and there is severe occlusion between the target and the similar object.

(1) *Basketball, Girl and Walking2*: in the Basketball, Girl and Walking2 sequences, target and occlusion object have similar appearance feature including structure and color. Staple uses color statistical feature to alleviate deformation, so the tracking result is easy to drift to occlusion object when the occlusion object has similar color feature with the target. Although the DAT algorithm also uses color statistical feature to resist deformation, but it does not consider the structural information of the target. It shows a poor performance on this video. Our tracker integrates ITSS to the Staple, which alleviates similar object interference, can resist deformation. In contrast, the Staple tracker does not obtain the spatial structure information of between the similar object and the target, and do not distinguish accurately the target and similar targets. Rest trackers rely too much on structural information and lead to drift when the target undergoes severe deformation.
(2) *Football and Girl*: in the Football sequence and the Girl sequence, the target is almost fully occluded by nearly the similar object. Thus, these trackers relying on structural feature mistakenly estimate the target location to the interference. In this case, our ITSS model is powerless because the structural features that the model relies on disappear. However, our tracker introduces a re-detection strategy which can effectively distinguish the target and nearly similar objects when the target appears again, then reconstruct the interference-target spatial structure. The experiment demonstrates that it is necessary to introduce the re-detection into our tracking framework to alleviate almost full occlusion.
(3) *Shaking1*: the target and interference have similar structure and color in the Shaking1 sequence. Compared to our algorithm, KCF and DSST exhibit varying degrees of drift because they have no ability to resist deformation. The Staple tracker the model, which can resist deformation by color statistical features, have no enough prior information for distinguishing target from similar objects. Our tracker combines effectively advantages of ITSS and Staple. When similarity interference occurs, using ITSS distinguishes the target and the interference. When the interference is removed, our algorithm switches to Staple. Therefore, our tracker can resist similarity interference as well as deformation.
(4) *Coupon*: the Coupon sequence is different from other sequences, where the target occludes the similar object. In addition, the target and the interference are obviously different in structural feature, but similar in color statistical feature in the sequence. Thus, except DAT, all other algorithms track the target robustly. Our tracking model not only gains the color feature of target appearance but also obtains its structure feature; therefore, our tracker gains satisfactory performance on this type of sequence.
(5) *Liquor*: there exist multiple structures similar targets in the Liquor sequence. In addition, the target is occluded repeatedly by different similar objects. Only our approach and Staple keep correct tracking throughout the tracking process. Staple utilizes the color difference between the target and other objects to distinguish them, while our approach uses ITSS constraint to discriminate the target from similar objects.
(6) *Bolt2 and Deer*: These two sequences are different from other sequences. The target is not occluded by a similar object, but the similar object is occluded by it. When the similar object gradually approaches the target, some tracker track the similar target incorrectly, e.g., DSST, while our tracker can use the learned ITSS constraint to correctly differentiate target and interference.

Overall, our approach provides promising results compared to the six other trackers on these sequences including similar object interference.

#### 3.2.2. Quantitative Evaluation

**Figure 3:**
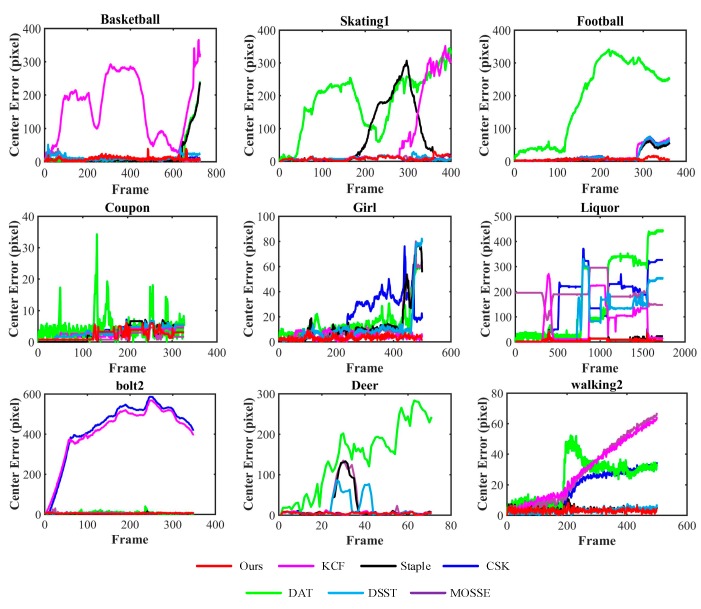
Frame-by-frame comparison of 7 trackers on 9 video sequences in terms of average center location errors (in pixels) in descending order. The horizontal axis represents the frame number, and the vertical axis represents the center location error. The smaller is the center location error, the better is the tracking accuracy.

**Figure 4:**
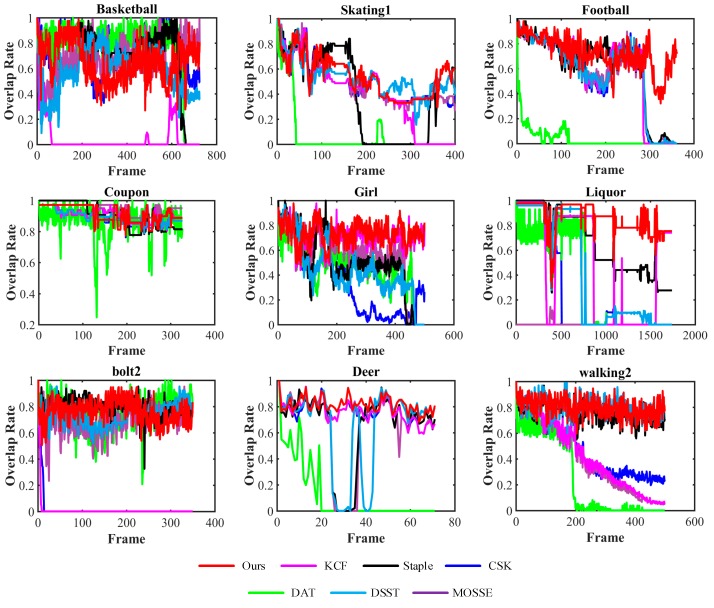
Frame-by-frame comparison of seven trackers on nine video sequences in terms of overlap ratio. The horizontal axis represents the frame number, and the vertical axis represents the overlap rate. The greater is the value, the better is the tracking accuracy.

In addition, we provide a quantitative comparison of our tracker and the six trackers on the nine challenging sequences mentioned above. Our quantitative evaluations with fame-by-frame comparison are two metrics: center location errors (CLE) and overlap ratio (OR). CLE is measured by the Euclidean distance between the ground-truth center location and the estimated target center location. OR is described as $\mathrm{OR}=S\left(B_{T}\cap B_{GT}\right)/S\left(B_{T}\cup B_{GT}\right)$, where $B_{GT}$ and $B_{T}\;$are the ground truth bounding box and the tracking bounding box, respectively, and S(*B*) is a function used to calculate the area of the bounding box *B*. We compare the CLE and VOR frame-by-frame on the nine sequences in Figure 3 and Figure 4, respectively. Generally, our method is comparable to the best performer on the sequence *Basketball*, *Skating1*, *Football*, *Coupon*, *Girl*, *Liquor*, *Bolt2*, *Deer* and *Walking2*. In particular, on the sequence *Basketball* and *Skating1*, our tracker slightly drifts in the 200th and 230th frames, respectively, due to in plane rotation of the target and deformation of the nearest similar object appear simultaneously.

Table 1 and Table 2 report the comparison of the average CLE and the average OR of seven trackers in the nine sequences mentioned above. Our approach achieves the best results in six of the nine sequences in average CLE, and achieves the best performance in seven of the nine sequences in the average OR. These results demonstrate that our approach obtains superior performance than the six other trackers in alleviating similar object interference. In *Coupon* and *Basketball* sequences, our tracker gains the second and the third biggest, respectively, which is very close to the best ones. The overall results present that the proposed approach significantly outperforms the others in average CLE and average OR.

Table 3 shows the time consumption. In the experiment, the main time consumption of our approach is embodied in three aspects: the Staple tracking model (basic tracker), the similarity target detection and the ITSS model. In the Staple tracking model, the main factor affecting the computation is the parameter of the HOG feature and the color histogram feature. In practice, the cell size of HOG features is set into 4×4 pixels. We set the area of the samples to $4^{2}$ times of the target area. Samples are multiplied by a Hann window. Then, samples are normalized to a fixed size by a 50×50 square, so that the fps is unaffected by the height and width of video sequence. Moreover, bin color histogram is set into 32×32×32. In the similarity target detection, time consumption of this part is similar with Staple model. In this part, the parameters of HOG feature and color histogram feature are similar to the parameters in Staple model. In the ITSS model, the time consumption is related to the number of nodes in the graph. We set that the max number of nodes in the graph to 8 and the fps to 24 when only running ITSS model. In practice, the number of nodes is controlled by w in Equation (1), and is usually less than 8. Moreover, our approach is switched dynamically between the baseline model and the constraint model by weight w in Equation (1). Thus, the time consumption of the approach should be between the Staple and the ITSS. We run our approach approximately for 35 frames per second on a computer with an Intel I7-4790 CPU (3.6 GHz) and 4 GB RAM. Therefore, our algorithm satisfies the real-time applications.

### 3.3. Comprehensive Performance Evaluation

To evaluate our tracker in comprehensive performance, we perform comprehensive evaluation using one-pass evaluation (OPE) on benchmark datasets [28]: OTB-2013 and OTB-2015. The OPE includes two scenarios: distance precision (DP) and overlap success rate (OSR). The threshold in DP is 20 pixels while OSR uses the area under curve (AUC) as the evaluation criterion. We compare our tracker called ITSS with state-of-art trackers including Staple [16], DLSSVM [29], MEEM [21], DSST [12], Struck [30], KCF [11], TLD [8], MOSSE [9], VTD [1], CSK [10], MIL [7] , and Frag [22].

**Figure 5:**
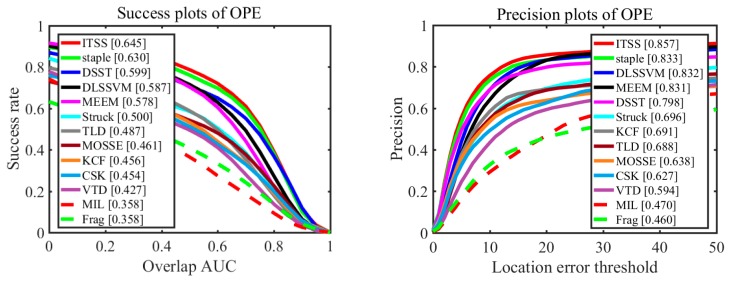
Distance precision and overlap success plots on entire OTB-2013 dataset using OPE.

**Figure 6:**
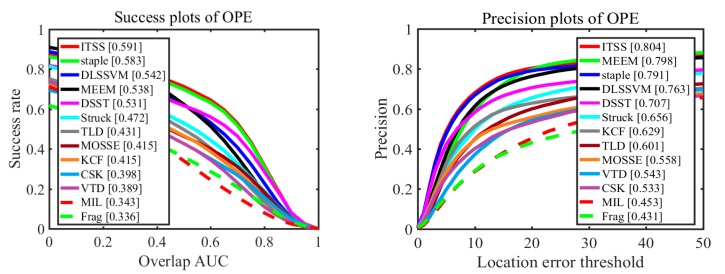
Distance precision and overlap success plots on entire OTB-2015 dataset using OPE.

We present the results of OTB-2013 and OTB-2015 in Figure 5 and Figure 6, respectively. Among these methods, our approach performs well with overall success (64.5% on OTB-2013 and 59.1% on OTB-2015) and precision plots (85.7% on OTB-2013 and 80.4% on OTB-2015). Moreover, our approach achieves higher performance than Staple tracker on these two datasets. Specifically, the OSR of the proposed approach is 1.5% and 0.8% higher than Staple tracker on these two datasets, respectively. The DP of our approach is 2.4% and 1.3% higher than Staple tracker on these two datasets, respectively. Because our approach integrates the advantages of Staple tracker and ITSS constraint, it would implicitly mitigate the weaknesses of HOG and color features, and resist similar object interference at the same time. In other words, the overall evaluation proves that our approach improves the performance (alleviating interference from similar object) of Staple algorithm and does not weaken its existing advantages.

Comparing Figure 5 with Figure 6, the performances of all trackers in Figure 5, including success plots of OPE and precision plots of OPE, are better than those in Figure 6, because those sequences reflecting performance of every tracker makes up a smaller proportion in OTB-2015 than in OTB-2013. Similarly, our algorithm also performs better in dataset OTB-2013 than in dataset OTB-2015.

## 4. Conclusions

In this paper, we propose a generic approach for alleviating similar object interference. The approach integrates the interference-target spatial structure (ITSS) constraint to existing CF tracking algorithm for improving the robustness of the algorithm when the target is severely occluded by a similar object. When a similar object exists around the target, the proposed ITSS constraint is online learning by using a minimum spanning tree model to optimize the structured SVM, which provides an effective strategy to identify the target and similar object when there is occlusion between them. Moreover, when the target is almost completely occluded by a similar object, we combine the target model and the interference model for re-detecting the missing target. The experimental results demonstrate that the proposed algorithm performs favorably against the state-of-the-art methods.

## Figures and Tables

**Table 1:** Comparison results in terms of average CLE (in pixels). The best three estimates are marked in red, blue, and green fonts in descending order.

Sequences	CSK	DAT	KCF	Staple	MOSSE	DSST	Ours
*Basketball*	**6.53**	17.72	**7.89**	16.89	162.21	10.92	**8.38**
*Coupon*	3.240	4.45	**1.57**	2.84	**2.80**	3.23	**2.28**
*Football*	16.19	189.31	**14.60**	**13.00**	16.86	15.76	**5.05**
*Girl*	19.34	15.25	11.92	13.12	**4.65**	**11.11**	**3.10**
*Liquor*	160.56	171.86	193.74	**8.68**	**71.75**	98.70	**5.20**
*Skating1*	**7.78**	182.62	**7.670**	70.62	66.86	8.33	**7.57**
*Bolt2*	429.40	6.630	6.370	**4.05**	414.48	**4.510**	**4.21**
*Deer*	**4.970**	143.90	21.16	19.73	**4.60**	16.66	**3.97**
*Walking2*	17.93	23.13	28.98	**3.43**	29.20	**2.950**	**2.88**
Average	73.99	83.87	32.66	16.93	85.93	19.13	4.73

**Table 2:** Comparison results in terms of average OR. The best three estimates are marked in red, blue, and green fonts in descending order.

Sequences	CSK	DAT	KCF	Staple	MOSSE	DSST	Ours
*Basketball*	**0.71**	**0.75**	0.65	**0.71**	0.05	0.58	**0.68**
*Coupon*	0.90	0.86	**0.940**	**0.900**	**0.91**	0.9	**0.91**
*Football*	0.56	0.04	0.56	**0.60**	**0.57**	**0.57**	**0.72**
*Girl*	0.38	0.47	**0.55**	0.50	**0.70**	0.44	**0.73**
*Liquor*	0.25	0.34	0.11	**0.660**	**0.48**	0.41	**0.76**
*Skating1*	**0.50**	0.07	0.49	0.410	0.40	**0.53**	**0.54**
*Bolt2*	0.02	**0.74**	0.69	**0.80**	0.01	**0.74**	**0.86**
*Deer*	**0.76**	0.12	0.64	**0.65**	**0.76**	**0.65**	**0.81**
*Walking2*	**0.47**	0.25	0.41	**0.77**	0.43	**0.80**	**0.80**
Average	0.51	0.40	0.55	0.67	0.48	0.62	0.75

**Table 3:** Comparing the Seven trackers on the nine sequences, the average time consumption of every tracker is shown in terms of fps.

Tracker	CSK	DAT	KCF	Staple	MOSSE	DSST	Ours
**FPS**	185	60	178	58	350	60	35
